# (η^5^-Carb­oxy­cyclo­penta­dien­yl)(η^7^-cyclo­hepta­trien­yl)manganese(I) hexa­fluorido­phosphate

**DOI:** 10.1107/S2414314623001074

**Published:** 2023-02-09

**Authors:** Reinhard Thaler, Klaus Wurst, Benno Bildstein

**Affiliations:** a Universität Innsbruck, Institut für Allgemeine, Anorganische und Theoretische Chemie, Innrain 80-82, 6020 Innsbruck, Austria; Vienna University of Technology, Austria

**Keywords:** tromancenium, manganese, cationic carb­oxy­lic acid, heteroleptic sandwich complexes, carb­oxy­lic acid, sandwich complexes, crystal structure

## Abstract

The title compound, [Mn(C_7_H_7_)(C_6_H_5_O_2_)]PF_6_ or [(Cht)Mn(Cp’CO_2_H)]PF_6_, with Cht = cyclo­hepta­trienyl and Cp’ = C_5_H_4_, is an air-stable, purple, heteroleptic, cationic sandwich complex with manganese in oxidation state +I and π-coordinating cyclo­hepta­trienyl and cyclo­penta­dienyl ligands. The latter ligand carries the carb­oxy­lic acid functionality. This ‘tromancenium-8-carb­oxy­lic acid’ with hexa­fluorido­phosphate as counter-ion represents a rare case of a cationic carb­oxy­lic acid. Structurally, this organometallic carb­oxy­lic acid displays the common motif of planar O*sp* ^2^⋯H—O*sp* ^3^/O*sp* ^3^—H⋯O*sp* ^2^ hydrogen-bonded carb­oxy­lic acid dimers with *anti*-oriented metallocenyl moieties, the cationic charge of which is balanced by octa­hedrally shaped hexa­fluorido­phosphate anions. Positional disorder is observed in the cyclo­hepta­trienyl ring and the PF_6_^−^ anion.

## Abstract 2

The ‘tromancenium-8-carb­oxy­lic acid’ entity with a hexa­fluorido­phosphate counter-ion represents a rare case of a cationic carb­oxy­lic acid.

## Graphical abstract

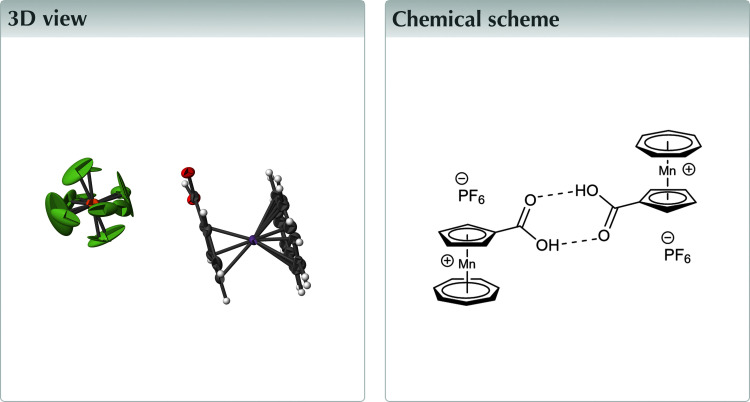

## Structure description

Cobaltocenium carb­oxy­lic acid hexa­fluorido­phosphate (Vanicek *et al.*, 2014 ▸) is a key compound for other monofunctionalized cobaltocenium salts and was synthesized starting from cobaltocenium by nucleophilic attack using (H_3_C)_3_SiC≡CLi, followed by hydride abstraction, silicon dissociation using NaF and oxidation to the desired carb­oxy­lic acid using KMnO_4_. As a result of the instability against nucleophiles of the parent compound tromancenium hexa­fluorido­phosphate (Basse *et al.*, 2021 ▸), the related title compound was synthesized by bypassing the use of carbon nucleophiles, whereby the carb­oxy­lic acid functionality was introduced as a masked methyl ester on its cymantrene precursor level. Photolysis of all three carbonyl ligands in presence of cyclo­hepta­trienyl, followed by oxidation with tritylium led to 8-carbometh­oxy tromancenium, the masked carb­oxy­lic acid (Basse *et al.*, 2021 ▸). Approaches for hydrolysis using aqueous NaOH led to complete decomposition, but inter­estingly the weaker base Na_2_CO_3_ led to hydrolysis without decomposition of the complex.

**Figure 1:**
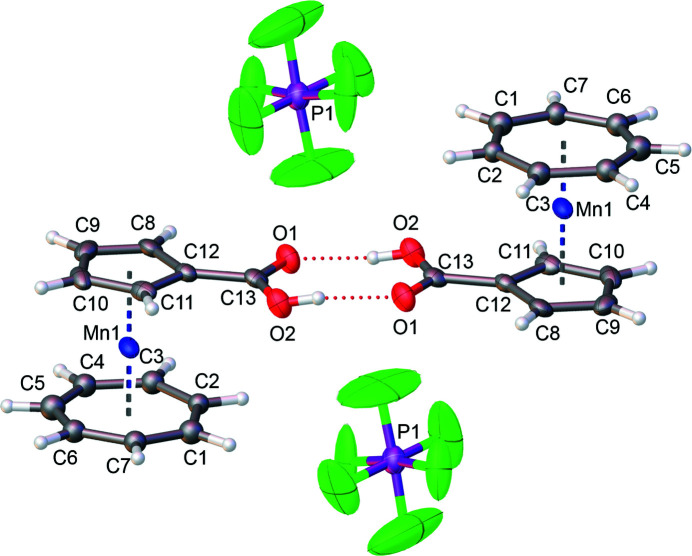
The mol­ecular entities of the title compound, with displacement ellipsoids drawn at the 50% probability level. The left cation and the anion at the bottom are related to their counterparts by inversion symmetry (symmetry operation −*x* + 1, −*y* + 1, −*z* + 1). For clarity, only one of the two positionally disordered parts of the cyclo­hepta­trienyl rings is shown. Likewise, for the disordered PF_6_^−^ anion, only the part with the higher occupancy is displayed.

The mol­ecular entities of the title compound are shown in Fig. 1 ▸. Positional disorder of the cyclo­hepta­trienyl ligand as well as of the PF_6_^−^ counter-ion was observed. The tromancenium carb­oxy­lic acid exists as a centrosymmetric dimer linked by mutual O*sp* ^2^⋯H—O*sp* ^3^/O*sp* ^3^—H⋯O*sp* ^2^ hydrogen bonds of the carb­oxy­lic acid moiety (Table 1 ▸), with tromanceniumyl in an *anti*-conformation to each other. The average Mn—C_Cp_ bond length of 2.09 Å is slightly longer than the average Mn—C_Cht_ bond length of 2.06 Å resulting from geometric reasons. The C12—C13 bond length of 1.482 (8) Å is typical for a carbon–carbon single bond. The C13—O1 bond length of 1.205 (10) Å is shorter than the C13—O2 bond length of 1.303 (10) Å, which is coherent with the expectations.

The comparable organometallic compound cobaltocenium carb­oxy­lic acid hexa­fluorido­phosphate (Vanicek *et al.*, 2014 ▸) shows an average Co—C(unsubstituted Cp) bond length of 2.02 Å and an average Co—C(substituted Cp) average bond length of 2.04 Å, which are slightly shorter than the average Mn—C_Cht/Cp_ bond lengths in the title compound. The C=O bond in cobaltocenium carb­oxy­lic acid is of the same length as the C—O bond, due to disorder.

**Figure 2:**
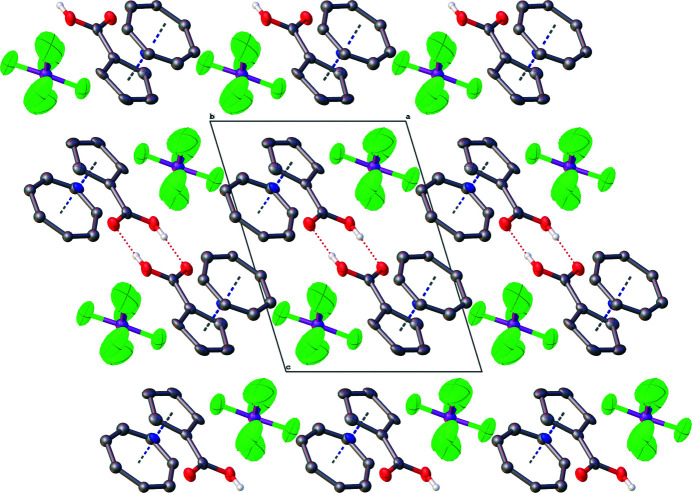
The packing along the crystallographic *b* axis, displaying alternating layers of tromancenium carb­oxy­lic acid dimers and hexa­fluorido­phosphate counter-ions.

We find typical bond lengths within the carb­oxy­lic acid moiety in the tromancenium system comparable to common organic carb­oxy­lic acids, but because of the cationic charge there are two counter-ions (PF_6_^−^), which fill the space within the packing of the dimers (Fig. 1 ▸). The packing along the crystallographic *b* axis displays alternating layers of tromancenium carb­oxy­lic acid dimers and hexa­fluorido­phosphate counter-ions. (Fig. 2 ▸).

## Synthesis and crystallization

A round-bottom flask was charged with 0.0563 g of 8-carbo­meth­oxy­tromancenium hexa­fluorido­phosphate (Basse *et al.*, 2021 ▸) (0.1359 mmol, 1 equiv) and dissolved in 10 ml of THF/water (1:1) before 0.266 ml of a saturated sodium carbonate solution were added. The mixture was stirred for 4 h and cooled to 273 K before 0.090 ml of an aqueous solution of HCl (37%_wt_) were added. The solvents were removed on a rotary evaporator and the crude material dried *in vacuo*. The product was dissolved in aceto­nitrile and filtered through a folded paper filter. Aceto­nitrile was removed on a rotary evaporator and the product was dried *in vacuo* giving pure 8-tromancenium carb­oxy­lic acid hexa­fluorido­phosphate in 92% yield (0.050 g, 0.1249 mmol). Single crystals were obtained by diffusion crystallization in aceto­nitrile out of diethyl ether at room temperature.

Properties: m.p.: 395.8 K dec. ^1^H NMR (400 MHz, CD_3_CN, p.p.m.) δ = 4.89 (pseudo-*t*, 2H, C10/C11 of Cp, *J*_1_ = 1.6 Hz, *J*_2_ = 2.0 Hz), 5.21 (pseudo-*t*, 2H, C9/C12 of Cp, *J* = 1.6 Hz), 6.93 (*s*, 7H, C1–7 of Cht); signal of CO_2_H not observed due to rapid exchange. ^13^C NMR (75 MHz, CD_3_CN, p.p.m.) δ = 78.6 (*ipso*-carbon of Cp), 79.4 (C10/C11 of Cp), 80.3 (C9/C12 of Cp), 99.0 (C1–7 of Cht), 156.4 (CO_2_H). ^55^Mn NMR (74 MHz, CD_3_CN, p.p.m.) δ = 529. IR (ATR, cm^−1^): 3000 (ν_O—H_ + ν_C—H_), 1696 (ν_C=O_), 1489, 1448, 1413, 1375 (ν_C—OH_ + ν_C=C_), 815 (ν_P—F_), 749 (δ_oop_,_*C*—H (Cp+Cht)_), 600, 554 (δ_oop_,_*O*—H_), 467, 437 (ν_Mn_). HRMS (ESI pos, *m*/*z*) 255.0211 ([*M* − PF_6_]^+^), calculated for C_13_H_12_O_2_Mn: 255.0212. UV–vis (CH_3_CN, [nm]) λ_max1_ = 283, λ_max2_ = 559. Cyclic voltammetry (CV): Δ*E*_1/2_ (Mn^+^/Mn^2+^) = 1.00 V *versus* ferrocene/ferrocenium^+^ (irreversible).

## Refinement

Crystal data, data collection and structure refinement details are summarized in Table 2 ▸. All probed crystals showed twinning by non-merohedry by rotation of 180° around the real axis [1 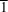 0]. The hydrogen atom attached to O2 was found from a difference-Fourier map and was refined isotropically with a distance restraint (*d* = 0.83 Å). A positional disorder in a ratio of 1:1 for the carbon atoms and attached hydrogen atoms of the seven-membered ring: C1–C7: C1*A*–C7*A* was considered; the corresponding carbon atoms were refined with isotropic displacement parameters. A further positional disorder of all fluorine atoms of the PF_6_^−^ anion was refined in ratio 45:55 for F1–F6:F1*A*—F6*A* with anisotropic displacement parameters. In an alternative model, the crystal structure was also refined in the non-centrosymmetric space group *P*1 with a new data set, for which *TWINABS* (Bruker, 2013 ▸) was used for absorption correction without merging Friedel pairs. This led to an ordered arrangement of two cyclo­hepta­trienyl rings and two PF_6_^−^ anions but unrealistic inter­actomic distances. The resulting Flack *x* parameter of 0.37 (8) in the *P*1 model and several remaining electron-density peaks between the carbon atoms of the two seven-membered rings clearly show that the disorder will be retained in the non-centrosymmetric space group. Hence, the latter was discarded and the centrosymmetric model used for final processing.

## Supplementary Material

Crystal structure: contains datablock(s) global, I. DOI: 10.1107/S2414314623001074/wm4180sup1.cif

Structure factors: contains datablock(s) I. DOI: 10.1107/S2414314623001074/wm4180Isup2.hkl

CCDC reference: 2239883

Additional supporting information:  crystallographic information; 3D view; checkCIF report

## Figures and Tables

**Table 1:** Hydrogen-bond geometry (Å, °)

*D*—H⋯*A*	*D*—H	H⋯*A*	*D*⋯*A*	*D*—H⋯*A*
O2—H2⋯O1^i^	0.83 (2)	1.81 (3)	2.638 (6)	173 (15)

Symmetry code: (i) 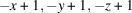 .

**Table 2:** Experimental details

Crystal data	
Chemical formula	[Mn(C_7_H_7_)(C_6_H_5_O_2_)]PF_6_
*M*_r_	400.14
Crystal system, space group	Triclinic, *P* 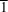
Temperature (K)	183
*a*, *b*, *c* (Å)	8.243 (8), 8.313 (7), 11.154 (12)
α, β, γ (°)	75.25 (3), 70.89 (2), 78.19 (4)
*V* (Å^3^)	692.2 (11)
*Z*	2
Radiation type	Mo *K*α
μ (mm^−1^)	1.14
Crystal size (mm)	0.12 × 0.11 × 0.04

Data collection	
Diffractometer	Bruker D8 QUEST PHOTON 100
Absorption correction	Multi-scan (*TWINABS*; Bruker, 2013 ▸)
*T*_min_, *T*_max_	0.779, 0.928
No. of measured, independent and observed [*I* > 2σ(*I*)] reflections	2349, 2349, 2066
(sin θ/λ)_max_ (Å^−1^)	0.595

Refinement	
*R*[*F* ^2^ > 2σ(*F* ^2^)], *wR*(*F* ^2^), *S*	0.053, 0.123, 1.17
No. of reflections	2349
No. of parameters	261
No. of restraints	1
H-atom treatment	H atoms treated by a mixture of independent and constrained refinement
Δρ_max_, Δρ_min_ (e Å^−3^)	0.52, −0.63

Computer programs: *APEX3* and *SAINT* (Bruker, 2013 ▸), *SHELXT* (Sheldrick, 2015 ▸) and *OLEX2* (Dolomanov *et al.*, 2009 ▸).

## full crystallographic data

### Crystal data

**Table:**

[Mn(C_7_H_7_)(C_6_H_5_O_2_)]PF_6_	*Z* = 2
*M**_r_* = 400.14	*F*(000) = 400
Triclinic, *P*1	*D*_x_ = 1.920 Mg m^−^^3^
*a* = 8.243 (8) Å	Mo *K*α radiation, λ = 0.71073 Å
*b* = 8.313 (7) Å	Cell parameters from 2453 reflections
*c* = 11.154 (12) Å	θ = 2.8–25.0°
α = 75.25 (3)°	µ = 1.14 mm^−^^1^
β = 70.89 (2)°	*T* = 183 K
γ = 78.19 (4)°	Plate, pink
*V* = 692.2 (11) Å^3^	0.12 × 0.11 × 0.04 mm

### Data collection

**Table:**

Bruker D8 QUEST PHOTON 100 diffractometer	2349 measured reflections
Radiation source: Incoatec Microfocus	2349 independent reflections
Multi layered optics monochromator	2066 reflections with *I* > 2σ(*I*)
Detector resolution: 10.4 pixels mm^-1^	θ_max_ = 25.0°, θ_min_ = 2.0°
φ and ω scans	*h* = −9→9
Absorption correction: multi-scan (*TWINABS*; Bruker, 2013)	*k* = −9→9
*T*_min_ = 0.779, *T*_max_ = 0.928	*l* = 0→13

### Refinement

**Table:**

Refinement on *F*^2^	Hydrogen site location: mixed
Least-squares matrix: full	H atoms treated by a mixture of independent and constrained refinement
*R*[*F*^2^ > 2σ(*F*^2^)] = 0.053	*w* = 1/[σ^2^(*F*_o_^2^) + 3.4169*P*] where *P* = (*F*_o_^2^ + 2*F*_c_^2^)/3
*wR*(*F*^2^) = 0.123	(Δ/σ)_max_ < 0.001
*S* = 1.17	Δρ_max_ = 0.52 e Å^−^^3^
2349 reflections	Δρ_min_ = −0.63 e Å^−^^3^
261 parameters	Extinction correction: SHELXL-2018/3 (Sheldrick, 2015), Fc^*^=kFc[1+0.001xFc^2^λ^3^/sin(2θ)]^-1/4^
1 restraint	Extinction coefficient: 0.0124 (18)

### Special details

**Table:**

Geometry. All esds (except the esd in the dihedral angle between two l.s. planes) are estimated using the full covariance matrix. The cell esds are taken into account individually in the estimation of esds in distances, angles and torsion angles; correlations between esds in cell parameters are only used when they are defined by crystal symmetry. An approximate (isotropic) treatment of cell esds is used for estimating esds involving l.s. planes.
Refinement. Refined as a 2-component twin by rotation of 180 degrees around [1-10]. Hydrogen at O2 was found and refined isotropically with bond restraint (d = 83pm). A positional disorder in ratio of 1:1 for the carbon atoms of the 7-mebered ring: C1-C7 : C1A-C7A were refined with isotropic displacement parameters for the carbon atoms. A further positional disorder of all flourine atoms of the PF6-anion was refined in ratio 45:55 F1-6:F1A-6A) with anisotropic displacement parameters. The structure was also refined in the non-centrosymmetric space group P1 with a new data set, using for absorption correction TWINABS without merging Friedel pairs. This led for the ordered structure to a Flack x parameter of 0.37 (8) and several rest-electron density peaks between the carbon atoms of the two 7-membered rings, clearly showing that the disorder will be retained in the non-centrosymmetric space group.

### Fractional atomic coordinates and isotropic or equivalent isotropic displacement parameters (Å^2^)

**Table:**

	*x*	*y*	*z*	*U*_iso_*/*U*_eq_	Occ. (<1)
Mn1	0.22529 (16)	0.26263 (16)	0.26587 (9)	0.0216 (3)	
O1	0.3477 (7)	0.5719 (7)	0.4112 (6)	0.0307 (14)	
O2	0.5540 (7)	0.3531 (7)	0.4093 (6)	0.0301 (14)	
H2	0.586 (19)	0.384 (17)	0.462 (12)	0.14 (6)*	
C8	0.2600 (11)	0.5254 (10)	0.1903 (8)	0.028 (2)	
H8	0.181572	0.618924	0.217709	0.034*	
C9	0.2654 (12)	0.4613 (12)	0.0877 (7)	0.033 (2)	
H9	0.195983	0.500705	0.030250	0.040*	
C10	0.3957 (13)	0.3253 (13)	0.0864 (6)	0.0316 (18)	
H10	0.427612	0.253126	0.026344	0.038*	
C11	0.4775 (10)	0.3062 (10)	0.1876 (8)	0.027 (2)	
H11	0.570779	0.224657	0.204514	0.033*	
C12	0.3915 (10)	0.4303 (10)	0.2523 (5)	0.0228 (13)	
C13	0.4334 (10)	0.4542 (11)	0.3656 (5)	0.0237 (13)	
P1	0.7659 (3)	0.8148 (3)	0.18491 (17)	0.0334 (5)	
C1	0.226 (3)	0.093 (3)	0.456 (2)	0.029 (6)*	0.5
H1	0.281926	0.065331	0.522456	0.035*	0.5
C2	0.094 (3)	0.233 (3)	0.4608 (17)	0.022 (4)*	0.5
H2A	0.078704	0.290191	0.528283	0.026*	0.5
C3	−0.019 (3)	0.303 (2)	0.382 (2)	0.027 (5)*	0.5
H3	−0.096616	0.400481	0.402022	0.032*	0.5
C4	−0.027 (3)	0.243 (3)	0.276 (2)	0.026 (5)*	0.5
H4	−0.112705	0.299714	0.234457	0.032*	0.5
C5	0.077 (3)	0.110 (3)	0.2296 (16)	0.031 (4)*	0.5
H5	0.054543	0.085937	0.158137	0.037*	0.5
C6	0.208 (3)	0.006 (2)	0.268 (2)	0.026 (5)*	0.5
H6	0.261298	−0.076884	0.216745	0.031*	0.5
C7	0.279 (2)	−0.005 (3)	0.367 (2)	0.025 (5)*	0.5
H7	0.375175	−0.088804	0.372033	0.030*	0.5
F1	0.709 (5)	0.665 (6)	0.294 (3)	0.21 (2)	0.45
F2	0.830 (6)	0.963 (6)	0.080 (3)	0.21 (2)	0.45
F3	0.832 (4)	0.675 (4)	0.116 (3)	0.133 (15)	0.45
F4	0.711 (5)	0.957 (4)	0.256 (4)	0.118 (15)	0.45
F5	0.599 (3)	0.877 (3)	0.130 (2)	0.086 (8)	0.45
F6	0.935 (4)	0.782 (5)	0.233 (3)	0.108 (12)	0.45
C1A	0.143 (3)	0.180 (3)	0.4718 (12)	0.019 (4)*	0.5
H1A	0.147945	0.211572	0.546571	0.022*	0.5
C2A	0.015 (3)	0.279 (2)	0.414 (2)	0.027 (5)*	0.5
H2AA	−0.050750	0.362336	0.461702	0.033*	0.5
C3A	−0.043 (3)	0.287 (3)	0.302 (2)	0.032 (5)*	0.5
H3A	−0.129422	0.374679	0.281677	0.039*	0.5
C4A	0.028 (2)	0.167 (2)	0.2234 (15)	0.014 (4)*	0.5
H4A	−0.018442	0.172677	0.154767	0.017*	0.5
C5A	0.164 (3)	0.039 (3)	0.2408 (16)	0.022 (4)*	0.5
H5A	0.201305	−0.027436	0.176362	0.026*	0.5
C6A	0.257 (3)	−0.013 (2)	0.333 (2)	0.026 (5)*	0.5
H6A	0.336415	−0.111631	0.321995	0.031*	0.5
C7A	0.256 (3)	0.051 (3)	0.435 (2)	0.031 (6)*	0.5
H7A	0.338231	0.000134	0.481243	0.037*	0.5
F1A	0.763 (3)	0.6505 (18)	0.3111 (12)	0.059 (4)	0.55
F2A	0.763 (2)	0.974 (2)	0.0597 (15)	0.081 (6)	0.55
F3A	0.848 (2)	0.705 (3)	0.0906 (16)	0.077 (8)	0.55
F4A	0.680 (3)	0.917 (3)	0.2822 (17)	0.062 (6)	0.55
F5A	0.590 (2)	0.775 (3)	0.1809 (16)	0.077 (6)	0.55
F6A	0.939 (3)	0.835 (3)	0.195 (2)	0.090 (9)	0.55

### Atomic displacement parameters (Å^2^)

**Table:**

	*U*^11^	*U*^22^	*U*^33^	*U*^12^	*U*^13^	*U*^23^
Mn1	0.0178 (6)	0.0261 (7)	0.0210 (5)	−0.0091 (3)	0.0015 (5)	−0.0093 (5)
O1	0.024 (3)	0.034 (3)	0.036 (3)	−0.001 (3)	−0.003 (2)	−0.020 (3)
O2	0.021 (3)	0.032 (3)	0.041 (4)	0.002 (3)	−0.009 (3)	−0.018 (3)
C8	0.033 (5)	0.021 (4)	0.031 (4)	−0.014 (4)	−0.005 (4)	−0.004 (4)
C9	0.036 (5)	0.042 (6)	0.022 (4)	−0.022 (4)	−0.009 (4)	0.004 (4)
C10	0.033 (5)	0.040 (6)	0.024 (3)	−0.023 (4)	0.005 (4)	−0.012 (4)
C11	0.023 (4)	0.025 (4)	0.027 (4)	−0.008 (4)	0.008 (3)	−0.009 (3)
C12	0.023 (4)	0.020 (4)	0.024 (3)	−0.009 (2)	0.000 (3)	−0.006 (3)
C13	0.019 (4)	0.023 (5)	0.030 (3)	−0.010 (2)	−0.004 (4)	−0.005 (4)
P1	0.0304 (13)	0.0398 (15)	0.0312 (9)	−0.0038 (7)	−0.0056 (10)	−0.0145 (11)
F1	0.13 (2)	0.27 (4)	0.18 (3)	−0.16 (3)	−0.03 (2)	0.13 (3)
F2	0.20 (4)	0.27 (4)	0.13 (2)	−0.12 (3)	−0.07 (2)	0.12 (2)
F3	0.21 (3)	0.065 (15)	0.18 (3)	0.085 (16)	−0.15 (3)	−0.091 (18)
F4	0.18 (4)	0.035 (11)	0.19 (3)	0.044 (15)	−0.13 (3)	−0.051 (15)
F5	0.056 (13)	0.15 (2)	0.084 (18)	0.031 (15)	−0.039 (13)	−0.081 (16)
F6	0.081 (17)	0.17 (3)	0.112 (17)	0.081 (17)	−0.065 (14)	−0.13 (2)
F1A	0.101 (13)	0.037 (7)	0.032 (5)	0.000 (6)	−0.011 (7)	−0.009 (5)
F2A	0.095 (13)	0.068 (9)	0.034 (7)	0.046 (9)	−0.008 (7)	0.012 (6)
F3A	0.035 (8)	0.16 (2)	0.045 (7)	−0.010 (10)	0.016 (6)	−0.078 (10)
F4A	0.048 (8)	0.101 (19)	0.041 (7)	0.035 (9)	−0.012 (6)	−0.060 (10)
F5A	0.035 (7)	0.149 (17)	0.060 (10)	−0.033 (11)	0.000 (7)	−0.048 (10)
F6A	0.028 (8)	0.093 (14)	0.17 (2)	−0.024 (8)	−0.015 (10)	−0.074 (14)

### Geometric parameters (Å, º)

**Table:**

Mn1—C10	2.044 (7)	P1—F3A	1.471 (16)
Mn1—C11	2.044 (8)	P1—F4A	1.459 (14)
Mn1—C12	2.093 (5)	P1—F5A	1.567 (17)
Mn1—C2	2.062 (16)	P1—F6A	1.511 (17)
Mn1—C3	2.02 (2)	C1—H1	0.9500
Mn1—C4	2.085 (19)	C1—C2	1.42 (2)
Mn1—C5	2.119 (15)	C1—C7	1.35 (3)
Mn1—C6	2.164 (19)	C2—H2A	0.9500
Mn1—C1A	2.139 (13)	C2—C3	1.43 (3)
Mn1—C2A	1.974 (19)	C3—H3	0.9500
Mn1—C3A	2.09 (2)	C3—C4	1.42 (3)
Mn1—C5A	2.130 (15)	C4—H4	0.9500
O1—C13	1.205 (10)	C4—C5	1.36 (3)
O2—H2	0.83 (2)	C5—H5	0.9500
O2—C13	1.303 (10)	C5—C6	1.35 (3)
C8—H8	0.9500	C6—H6	0.9500
C8—C9	1.365 (11)	C6—C7	1.39 (3)
C8—C12	1.463 (12)	C7—H7	0.9500
C9—H9	0.9500	C1A—H1A	0.9500
C9—C10	1.390 (11)	C1A—C2A	1.42 (3)
C10—H10	0.9500	C1A—C7A	1.33 (3)
C10—C11	1.456 (12)	C2A—H2AA	0.9500
C11—H11	0.9500	C2A—C3A	1.46 (3)
C11—C12	1.354 (10)	C3A—H3A	0.9500
C12—C13	1.482 (8)	C3A—C4A	1.41 (3)
P1—F1	1.53 (3)	C4A—H4A	0.9500
P1—F2	1.52 (4)	C4A—C5A	1.40 (3)
P1—F3	1.47 (2)	C5A—H5A	0.9500
P1—F4	1.50 (3)	C5A—C6A	1.41 (3)
P1—F5	1.62 (2)	C6A—H6A	0.9500
P1—F6	1.60 (3)	C6A—C7A	1.37 (3)
P1—F1A	1.691 (15)	C7A—H7A	0.9500
P1—F2A	1.665 (16)		
			
C10—Mn1—C12	65.1 (2)	F3A—P1—F2A	87.6 (10)
C10—Mn1—C2	164.5 (5)	F3A—P1—F5A	85.5 (10)
C10—Mn1—C4	117.8 (6)	F3A—P1—F6A	92.2 (12)
C10—Mn1—C5	101.4 (5)	F4A—P1—F1A	86.2 (11)
C10—Mn1—C6	100.3 (6)	F4A—P1—F2A	94.4 (11)
C10—Mn1—C1A	157.0 (6)	F4A—P1—F3A	177.3 (13)
C10—Mn1—C3A	124.2 (7)	F4A—P1—F5A	92.8 (12)
C10—Mn1—C5A	97.9 (5)	F4A—P1—F6A	89.3 (13)
C11—Mn1—C10	41.7 (3)	F5A—P1—F1A	88.6 (9)
C11—Mn1—C12	38.2 (3)	F5A—P1—F2A	89.4 (10)
C11—Mn1—C2	124.2 (6)	F6A—P1—F1A	86.1 (12)
C11—Mn1—C4	159.5 (6)	F6A—P1—F2A	95.9 (13)
C11—Mn1—C5	133.1 (7)	F6A—P1—F5A	174.1 (13)
C11—Mn1—C6	109.6 (6)	Mn1—C1—H1	143.0
C11—Mn1—C1A	115.6 (5)	C2—C1—Mn1	64.4 (11)
C11—Mn1—C3A	161.7 (6)	C2—C1—H1	117.0
C11—Mn1—C5A	116.9 (6)	C7—C1—Mn1	73.0 (12)
C12—Mn1—C5	166.0 (5)	C7—C1—H1	117.0
C12—Mn1—C6	145.1 (7)	C7—C1—C2	126 (2)
C12—Mn1—C1A	99.2 (3)	Mn1—C2—H2A	136.4
C12—Mn1—C5A	154.9 (7)	C1—C2—Mn1	77.3 (12)
C2—Mn1—C12	99.6 (5)	C1—C2—H2A	114.3
C2—Mn1—C4	75.8 (9)	C1—C2—C3	131.4 (18)
C2—Mn1—C5	94.1 (6)	C3—C2—Mn1	67.9 (10)
C2—Mn1—C6	91.1 (8)	C3—C2—H2A	114.3
C3—Mn1—C10	146.6 (8)	Mn1—C3—H3	134.5
C3—Mn1—C11	153.7 (7)	C2—C3—Mn1	71.1 (11)
C3—Mn1—C12	115.8 (6)	C2—C3—H3	116.6
C3—Mn1—C2	41.0 (9)	C4—C3—Mn1	72.3 (11)
C3—Mn1—C4	40.5 (9)	C4—C3—C2	126.8 (18)
C3—Mn1—C5	73.1 (9)	C4—C3—H3	116.6
C3—Mn1—C6	94.0 (7)	Mn1—C4—H4	138.4
C4—Mn1—C12	144.2 (7)	C3—C4—Mn1	67.2 (11)
C4—Mn1—C5	37.6 (9)	C3—C4—H4	117.4
C4—Mn1—C6	70.6 (7)	C5—C4—Mn1	72.5 (10)
C5—Mn1—C6	36.6 (8)	C5—C4—C3	125.2 (19)
C2A—Mn1—C10	159.3 (8)	C5—C4—H4	117.4
C2A—Mn1—C11	145.5 (7)	Mn1—C5—H5	139.1
C2A—Mn1—C12	111.0 (6)	C4—C5—Mn1	69.9 (10)
C2A—Mn1—C1A	40.0 (8)	C4—C5—H5	114.6
C2A—Mn1—C3A	42.1 (9)	C6—C5—Mn1	73.5 (11)
C2A—Mn1—C5A	91.4 (7)	C6—C5—C4	130.9 (15)
C3A—Mn1—C12	132.5 (7)	C6—C5—H5	114.6
C3A—Mn1—C1A	78.8 (8)	Mn1—C6—H6	139.1
C3A—Mn1—C5A	71.9 (9)	C5—C6—Mn1	69.9 (10)
C5A—Mn1—C1A	90.7 (6)	C5—C6—H6	113.6
C13—O2—H2	116 (10)	C5—C6—C7	132.9 (17)
Mn1—C8—H8	126.3	C7—C6—Mn1	74.9 (12)
C9—C8—Mn1	73.6 (5)	C7—C6—H6	113.6
C9—C8—H8	124.6	Mn1—C7—H7	139.2
C9—C8—C12	110.8 (8)	C1—C7—Mn1	71.8 (12)
C12—C8—Mn1	67.0 (4)	C1—C7—C6	127 (2)
C12—C8—H8	124.6	C1—C7—H7	116.7
Mn1—C9—H9	128.9	C6—C7—Mn1	68.4 (11)
C8—C9—Mn1	70.2 (5)	C6—C7—H7	116.7
C8—C9—H9	127.9	Mn1—C1A—H1A	138.4
C8—C9—C10	104.3 (8)	C2A—C1A—Mn1	63.7 (9)
C10—C9—Mn1	64.4 (5)	C2A—C1A—H1A	115.7
C10—C9—H9	127.9	C7A—C1A—Mn1	78.2 (10)
Mn1—C10—H10	120.6	C7A—C1A—H1A	115.7
C9—C10—Mn1	77.8 (6)	C7A—C1A—C2A	128.7 (13)
C9—C10—H10	123.9	Mn1—C2A—H2AA	136.7
C9—C10—C11	112.2 (8)	C1A—C2A—Mn1	76.2 (10)
C11—C10—Mn1	69.1 (4)	C1A—C2A—H2AA	111.3
C11—C10—H10	123.9	C1A—C2A—C3A	137.5 (18)
Mn1—C11—H11	122.2	C3A—C2A—Mn1	73.1 (11)
C10—C11—Mn1	69.2 (4)	C3A—C2A—H2AA	111.3
C10—C11—H11	127.6	Mn1—C3A—H3A	135.3
C12—C11—Mn1	72.9 (4)	C2A—C3A—Mn1	64.8 (10)
C12—C11—C10	104.8 (8)	C2A—C3A—H3A	119.5
C12—C11—H11	127.6	C4A—C3A—Mn1	74.1 (11)
C8—C12—Mn1	72.9 (4)	C4A—C3A—C2A	120.9 (18)
C8—C12—C13	129.8 (8)	C4A—C3A—H3A	119.5
C11—C12—Mn1	68.9 (4)	Mn1—C4A—H4A	141.3
C11—C12—C8	107.9 (5)	C3A—C4A—Mn1	67.5 (10)
C11—C12—C13	122.3 (9)	C3A—C4A—H4A	118.2
C13—C12—Mn1	123.3 (4)	C5A—C4A—Mn1	69.3 (10)
O1—C13—O2	124.5 (5)	C5A—C4A—C3A	123.7 (17)
O1—C13—C12	114.5 (8)	C5A—C4A—H4A	118.2
O2—C13—C12	121.0 (8)	Mn1—C5A—H5A	137.0
F1—P1—F5	102.7 (18)	C4A—C5A—Mn1	72.6 (10)
F1—P1—F6	84.7 (19)	C4A—C5A—H5A	113.1
F2—P1—F1	177 (2)	C4A—C5A—C6A	133.9 (17)
F2—P1—F5	80.2 (17)	C6A—C5A—Mn1	74.1 (10)
F2—P1—F6	92 (2)	C6A—C5A—H5A	113.1
F3—P1—F1	79 (2)	Mn1—C6A—H6A	143.0
F3—P1—F2	102 (2)	C5A—C6A—Mn1	68.1 (10)
F3—P1—F4	176 (2)	C5A—C6A—H6A	113.3
F3—P1—F5	94.4 (15)	C7A—C6A—Mn1	74.9 (12)
F3—P1—F6	91.3 (16)	C7A—C6A—C5A	133.3 (19)
F4—P1—F1	102 (2)	C7A—C6A—H6A	113.3
F4—P1—F2	77 (2)	Mn1—C7A—H7A	139.5
F4—P1—F5	89.5 (17)	C1A—C7A—Mn1	67.0 (10)
F4—P1—F6	84.7 (18)	C1A—C7A—C6A	121.7 (19)
F6—P1—F5	171.4 (17)	C1A—C7A—H7A	119.2
F2A—P1—F1A	178.0 (10)	C6A—C7A—Mn1	69.6 (12)
F3A—P1—F1A	91.8 (10)	C6A—C7A—H7A	119.2
			
Mn1—C8—C9—C10	−55.6 (5)	C10—C11—C12—C13	179.1 (5)
Mn1—C8—C12—C11	60.3 (4)	C11—C12—C13—O1	178.7 (6)
Mn1—C8—C12—C13	−119.5 (6)	C11—C12—C13—O2	−1.5 (9)
Mn1—C9—C10—C11	−61.1 (5)	C12—C8—C9—Mn1	56.9 (5)
Mn1—C10—C11—C12	−64.8 (5)	C12—C8—C9—C10	1.3 (9)
Mn1—C11—C12—C8	−62.9 (4)	C1—C2—C3—Mn1	−47.6 (19)
Mn1—C11—C12—C13	116.9 (5)	C1—C2—C3—C4	1 (3)
Mn1—C12—C13—O1	−96.7 (8)	C2—C1—C7—Mn1	38.6 (18)
Mn1—C12—C13—O2	83.2 (8)	C2—C1—C7—C6	−5 (3)
Mn1—C1—C2—C3	44.5 (18)	C2—C3—C4—Mn1	−48.5 (17)
Mn1—C1—C7—C6	−43.4 (18)	C2—C3—C4—C5	−3 (3)
Mn1—C2—C3—C4	49.0 (17)	C3—C4—C5—Mn1	−44.0 (17)
Mn1—C3—C4—C5	45.9 (17)	C3—C4—C5—C6	0 (3)
Mn1—C4—C5—C6	44.1 (16)	C4—C5—C6—Mn1	−43.0 (15)
Mn1—C5—C6—C7	44 (2)	C4—C5—C6—C7	1 (3)
Mn1—C6—C7—C1	44.6 (19)	C5—C6—C7—Mn1	−43 (2)
Mn1—C1A—C2A—C3A	45 (2)	C5—C6—C7—C1	2 (4)
Mn1—C1A—C7A—C6A	−44.9 (17)	C7—C1—C2—Mn1	−41.4 (19)
Mn1—C2A—C3A—C4A	50.5 (16)	C7—C1—C2—C3	3 (4)
Mn1—C3A—C4A—C5A	42.6 (15)	C1A—C2A—C3A—Mn1	−46 (2)
Mn1—C4A—C5A—C6A	45.6 (18)	C1A—C2A—C3A—C4A	5 (3)
Mn1—C5A—C6A—C7A	40 (2)	C2A—C1A—C7A—Mn1	41.6 (12)
Mn1—C6A—C7A—C1A	43.9 (16)	C2A—C1A—C7A—C6A	−3 (2)
C8—C9—C10—Mn1	59.4 (5)	C2A—C3A—C4A—Mn1	−46.6 (15)
C8—C9—C10—C11	−1.8 (10)	C2A—C3A—C4A—C5A	−4 (3)
C8—C12—C13—O1	−1.5 (9)	C3A—C4A—C5A—Mn1	−41.9 (15)
C8—C12—C13—O2	178.3 (6)	C3A—C4A—C5A—C6A	4 (3)
C9—C8—C12—Mn1	−60.7 (6)	C4A—C5A—C6A—Mn1	−45.1 (18)
C9—C8—C12—C11	−0.4 (7)	C4A—C5A—C6A—C7A	−5 (4)
C9—C8—C12—C13	179.8 (6)	C5A—C6A—C7A—Mn1	−38 (2)
C9—C10—C11—Mn1	66.4 (6)	C5A—C6A—C7A—C1A	6 (3)
C9—C10—C11—C12	1.6 (8)	C7A—C1A—C2A—Mn1	−46.5 (14)
C10—C11—C12—Mn1	62.2 (4)	C7A—C1A—C2A—C3A	−2 (3)
C10—C11—C12—C8	−0.7 (6)		

### Hydrogen-bond geometry (Å, º)

**Table:**

*D*—H···*A*	*D*—H	H···*A*	*D*···*A*	*D*—H···*A*
O2—H2···O1^i^	0.83 (2)	1.81 (3)	2.638 (6)	173 (15)

Symmetry code: (i) −*x*+1, −*y*+1, −*z*+1.
